# Downregulation of DEAD-box helicase 21 (DDX21) inhibits proliferation, cell cycle, and tumor growth in colorectal cancer via targeting cell division cycle 5-like (CDC5L)

**DOI:** 10.1080/21655979.2021.2011636

**Published:** 2021-12-13

**Authors:** Kai Wang, Baosong Li, Peng Fan, Xiang Ren, Hong Jiang

**Affiliations:** Department of Colorectal and Anal Surgery, Binzhou Medical University Hospital, Binzhou, Shandong, P.R. China

**Keywords:** DDX21, CDC5L, colorectal cancer, proliferation, cell cycle arrest

## Abstract

Identification of novel anti-tumor target is crucial for cancer diagnosis, prognosis, and therapeutic strategy. The study aimed to explore the roles and interaction of DEAD-box helicase 21 (DDX21) and cell division cycle 5-like (CDC5L) in colorectal cancer (CRC) progression. Levels of DDX21 and CDC5L were detected in colorectal cancer cell lines by RT-qPCR and Western blot assay. The role of DDX21 and CDC5L on the cell proliferation, cell cycle and tumor growth were evaluated both *in vitro* and *in vivo*. The interaction of DDX21 and CDC5L was predicted by The STRING publicly available data and verified by immunoprecipitation. The results showed that DDX21 was dramatically upregulated in colorectal cancer cells. *In vivo* and *in vitro* experiments revealed that downregulation of DDX21 suppressed colorectal cancer cell proliferation, colony formation, cell cycle development, and tumor growth, while overexpression of CDC5L reversed the suppressive effects of DDX21 silencing. Furthermore, DDX21 interacted with CDC5L to exert the tumor-promoting effects in CRC. In summary, the data indicate a novel role for DDX21/CDC5L in the development of CRC, which enrich the therapeutic strategy for CRC.

## Introduction

Colorectal cancer (CRC) is a widespread human cancer and the incidence in China accounted for 10% of all diagnosed cancer cases in 2009 [1]. The diet and habits are the main factors of CRC, which leads to the increasing incidence of CRC in Asia and Africa [2]. Nowadays, the combination of chemotherapy and targeted agents has built up the survival time of patients with CRC, but the 5-year survival rate is still unsatisfied [3]. Some studied have reported that targeting stages in cell cycle process enhances tumor therapy effectiveness [4].

DEAD-box helicase 21 (DDX21) codes proteins that characterized as motif Glu-Ala-Asp (DEAD) box proteins are putative RNA helicases [5]. It has been reported that consumption of DDX21 prominently decreased ribosome RNA levels (18S and 28S) in different cell types [6]. DDX21 has been reported to interact with mitotic regulator serine/threonine phosphoprotein phosphatase (PP)1 and oncoprotein DEK regulating the tumor progression [7,8]. DDX21 is expressed highly in different cancer types such as breast cancer [9]. Clinical data have shown that DDX21 is a potential colorectal biomarker [10]. However, the detailed molecular mechanisms of DDX21 in CRC still rarely investigated.

Cell division cycle 5-like (CDC5L) is a key mediator for G2 stage and mitotic entry. CDC5L is also highly expressed in some human tumors including prostate cancer and hepatocellular carcinoma [11]. CDC5L protein as a cell cycle regulator regulates G2/M transition in gliomas [12]. Nonetheless, silence of CDC5L in tumor cells drastically induced mitotic arrest and DNA damage [13]. CDC5L interacts with ataxia-telangiectasia and Rad3-related (ATR), the cell-cycle checkpoint kinase, which mediates the DNA damage, such as activation of cell-cycle checkpoints (checkpoint kinase 1 and cell cycle checkpoint protein Rad 17) and apoptosis [14]. A research reveals that CDC5L as a key promoter of CRC cells regulates cell growth and migration both *in vivo* and *in vitro* [15].

According to String online prediction, DDX21 may be identified to interact with CDC5L. In this study, we aims to investigate the role of DDX21 in CRC progression and its possible mechanism, and we hypothesized that DDX21 plays a tumor-promoting role in CRC by binding to CDC5L. In this study, we found that DDX21 expressed highly in colorectal cancer cell lines and downregulation of DDX21 is crucial to proliferation and cell cycle arrest. We supposed that DDX21 interacts with CDC5L to regulate cell proliferation and cell cycle arrest.

## Materials and methods

### Cell lines

Human CRC cell lines, SW480, SW620, HT-29 and HCT116 and normal human colonic epithelial cells NCM460 were obtained from American Type Culture Collection (ATCC, USA). CRC cell lines were cultured in RPMI-1640 medium (Gibco) supplemented with 10% fetal bovine serum (Gibco) at 37°C with 5% CO_2_.

### Cell transfection

DDX21-specific shRNA (shRNA-DDX21-1/2) and corresponding control (shRNA-NC), CDC5L-specific pcDNA overexpression vector (CDC5L) and corresponding negative control (NC) were completed by Shanghai Genechem Co., Ltd., China. These recombinants were transfected into SW480 cells using Lipofectamine 2000 reagent (Invitrogen, USA) according to the manufacturer’s instructions [16]. After 48 h transfection, cells were collected for subsequent experiments.

*RT-qPCR*. TRIzol reagent (Sigma) was used to isolate total RNA from cells. RNA was reversed to cDNA using High Capacity RNA-to-cDNA Kit (Applied Biosystems). PCR reactions were conducted in an ABI PRISM 7900 Real-Time system (Applied Biosystems, Foster City, CA, USA) with AceQ Universal SYBR qPCR Master Mix (Vazyme, China). The primer sequences for PCR are presented as below: DDX21, forward 5ʹ-GAGGAGCCATCTCAAAATGACA-3ʹ and reverse 5ʹ-GGGTTACAGTCCGGTTCAGG-3ʹ; CDC5L, forward 5ʹ-GCAGCGGTA-ATGAAATATGGGA-3ʹ and reverse 5ʹ-CAATGATTGGAGCAATGGTCCT-3ʹ; GAPDH, forward 5ʹ-GGGAAACTGTGGCGTGAT-3′ and reverse 5′-GAGTGGGTGTCGCTGTTGA-3′. The relative mRNA level was normalized with GAPDH by 2^−ΔΔCt^ method [17].

*Proliferation assay*.

CCK-8

Cell viability was measured by Cell-counting assay kit-8 (CCK-8; Abcam) [18]. SW480 (shRNA-DDX21) cells or SW480 (shRNA-DDX21-pcDNA3.1-CDC5L) cells with concentration of 5 × 10^3^ /well were seeded in 96-well plates and incubated for 24, 48 and 72 hours, respectively. After incubation, the absorbance reading of each well was detected with microplate reader at 450 nm with Microplate Reader (Bio-Rad, La Jolla, CA, USA).

### Colony formation assay

SW480 (shRNA-DDX21) cells or SW480 (shRNA-DDX21-pcDNA3.1-CDC5L) cells with 1 × 10^3^/well were seeded in 6-well plates. After incubated at 37°C for 14 days, cells were fixed with 4% paraformaldehyde and stained with 0.1% crystal violet. The number of colony was counted by a light microscope (Olympus Corp, Japan). The colony containing more than 50 cells was identified as a colony. Each group was replicated for five times [19].

### Immunofluorescence

SW480 cells were seeded on coverslips (coated with poly-L-lysine, Thermo Fisher Scientific) in 12 well plate. After cells subsequently fixed with 4% paraformaldehyde for 10 min, cells were permeabilized using 0.5% Triton X-100 in PBS for 10 min. 5% nonfat milk was used to block the cells and subsequently incubated with Ki-67 antibody (ab197234, Abcam) for 1 h at room temperature. Mounting Medium with DAPI (ab104139, Abcam) was used to stain the nuclei. Images were obtained by fluorescence microscope (Olympus, Japan) and analyzed by image J software [20].

*Cell cycle analysis*. Cell cycle arrest was analyzed by flow cytometry protocol l [21]. Cells were collected and fixed with 70% ethanol at 4°C overnight. Subsequently, cells were stained with FxCycle PI/RNase Staining solution (Invitrogen) and detected immediately with a BD Accuri C6 flow cytometer (BD Biosciences, USA).

### Immunoprecipitation

Cells were lysed in immunoprecipitation buffer (50 mM HEPES pH 7.6, 150 mM NaCl, 5 mM EDTA, 0.1% NP-40) and centrifuged for 10 min at 15,000 g to remove particulate material. Then the cell lysates were incubated with anti-DDX21 and Protein AG Magnetic Beads (Bimake, Selleck Chemical, Houston, TX, USA) according to the manufacturer’s instructions at 4°C [22].

### Tumor xenograft mouse model

All animal experiments were approved by the Animal Research Ethics Committee of the Binzhou Medical University Hospital and were investigated according to Guide for the Care and Use of Laboratory Animals guidelines prepared by the National Academy of Sciences and published by the National Institutes of Health. Female BALB/c nude mice (six-week-old) was injected with SW480 cells (5 × 10^6^). Mice were randomized into four groups (n = 3): control, shRNA-DDX21, shRNA-DDX21-NC, shRNA-DDX21-CDC5L. The tumor volume and body weight were examined every 5 days. The tumor volume was calculated using the formula: volume = (length × width^2^)/2. Body weight was measured before mice sacrifice and tumors were harvested to weight [23].

### Immunohistochemistry

Tumor tissues were fixed in 4% paraformaldehyde, dehydrated, embedded in paraffin wax, and then cut into 5 µm sections. Sample was incubated with proteinase K for 30 minutes to allow antigen retrieval. The sections were blocked with 10% goat serum for 30 min at room temperature and were incubated with Ki67 (1:200, ab16667) antibodies at 4°C overnight. Then, the sample was cultured with secondary antibody for 1 h at room temperature, Thereafter, section will be exposed to a freshly prepared diaminobenzidine for 4‐6 minutes and stained with hematoxylin for 15 seconds [24].

### Western blotting

Total proteins were extracted using lysis buffer (Beyotime, China) [25]. Proteins were separated by 10% SDS-PAGE and transferred to polyvinylidene difluoride (PVDF) membrane. The primary antibodies, DDX21 (#ab115178, Abcam), CDC5L (#ab129114, Abcam), Cyclin B (#ab32053, Abcam), CDC2 (#4539, Cell Signaling Technology), MMP2 (#40,994, Cell-signaling Technology), MMP9 (#13,667, Cell Signaling Technology), and GAPDH (#5174, Cell Signaling Technology) were incubated with PVDF membrane, followed by horseradish peroxidase-conjugated anti-Rabbit IgG secondary antibodies (#ab205718, Abcam). The protein bands were stained using ECL, imaged using iBright CL750 Imaging System (Thermo Fisher Scientific) and were analyzed by Image J software.

### Statistical analysis

The date in graphs show three independent experiments with mean ± standard deviations. The differences between two groups using Student's *t*-test and in multiple groups using one-way ANOVA followed by Tukey’s post hoc test. The significant difference was set at P < 0.05.

## Results

In this study, we explored the biological role of DDX21 and the potential mechanism in CRC *in vivo* and *in vitro*. The data revealed that DDX21 was upregulated in CRC cells. DDX21 silencing suppressed the proliferation and promoted cell cycle arrest in SW480 cells and inhibited tumor growth in mice model. Mechanistic investigations showed that DDX21 targets CDC5L. Rescue experiments revealed that CDC5L reversed the effects of DDX21 silencing on proliferation, cell cycle and tumor growth.

## DDX21 was highly expressed in CRC cell lines

**Figure 1.**
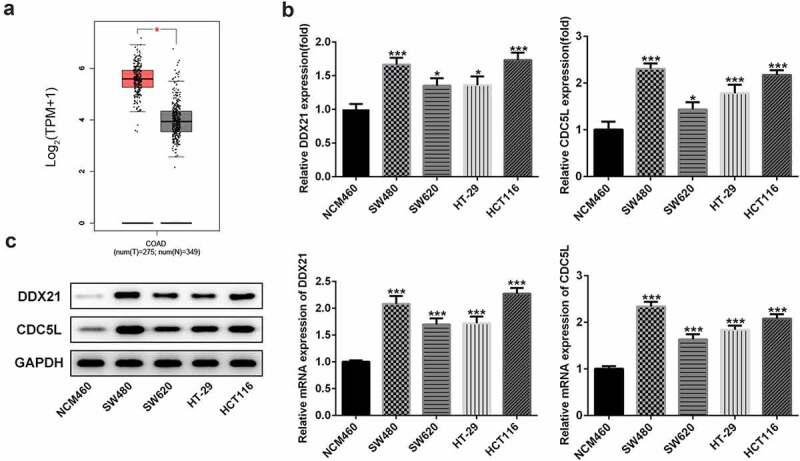
Expressions of DDX21 and CDC5L in CRC cell lines. (a) Analysis of GEPIA dataset to determine the expression pattern of DDX21 in CRC. The expression of DDX21 and CDC5L was assessed by RT-PCR (b) and Western blots (c) in CRC cell lines and normal control cells (*p < 0.05, **p < 0.01, ***p < 0.001 vs. NCM460)

To explore the role of DDX21 in CRC, we first detected expressions of DDX21 in CRC. The GEPIA database (http://gepia.cancer-pku.cn/) showed that DDX21 was significantly upregulated in tissues with CRC (Figure 1a). Then, RT-qPCR and western wlotting results revealed that level of DDX21 was dramatically increased in CRC cell lines especially in SW480 cells (Figure 1b,C). Thus, SW480 cells were used in the following experiments. The data indicate that DDX21 might be associated with CRC progression.

## DDX21 silencing repressed cell proliferation in SW480 cells

**Figure 2.**
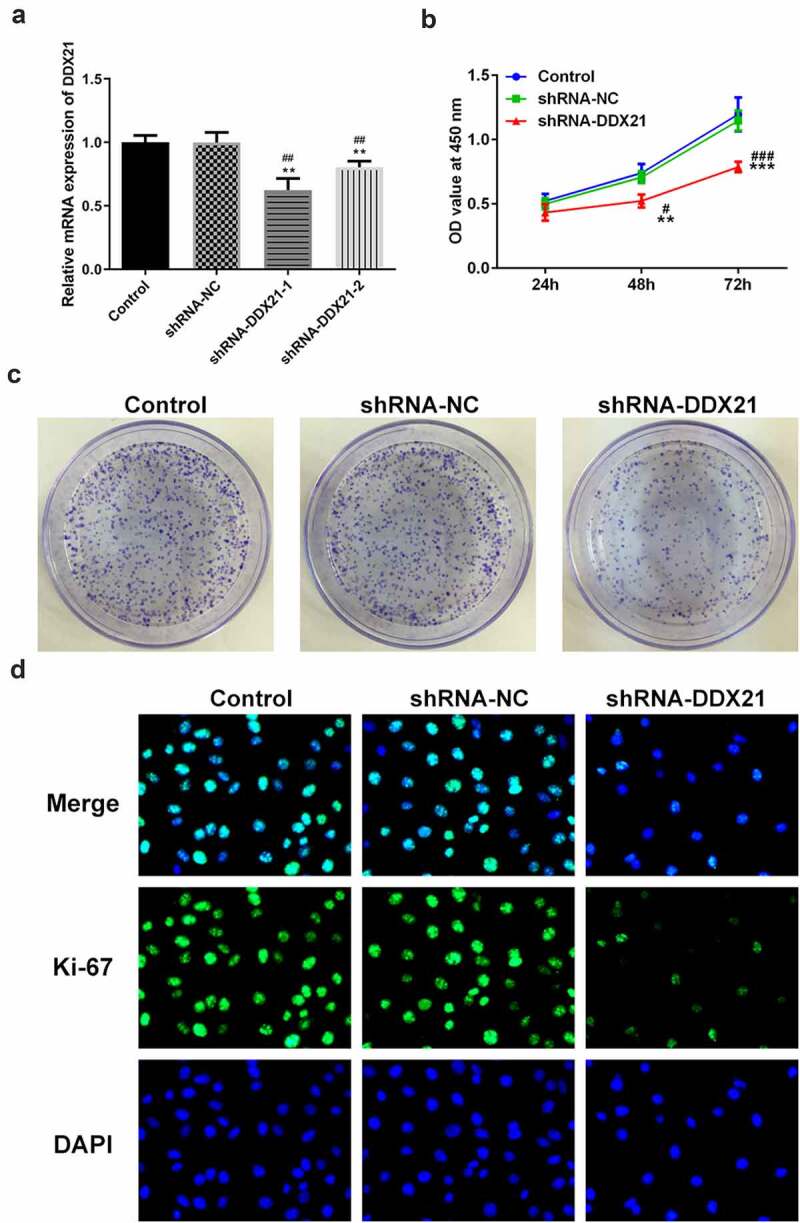
DDX21 knockdown inhibits cell proliferation in SW480 cells. (a)The expression of DDX21 in SW480 (wild type), SW480 (shRNA-NC; shRNA negative control) and SW480 (shRNA-DDX21) cells was detected by RT-PCR (**p < 0.01 vs. control, ^##^p < 0.01 vs. shRNA-NC). (b) The cell viability was determined by CCK-8 (**p < 0.01, ***p < 0.001 vs. control; ^#^p < 0.05, ^###^p < 0.001 vs. shRNA-NC). (c) The proliferation of SW480 cells in different groups was detected by clonal formation assay. (d) ki-67 expression and location in different groups was evaluated by immunofluorescence

**Figure 3.**
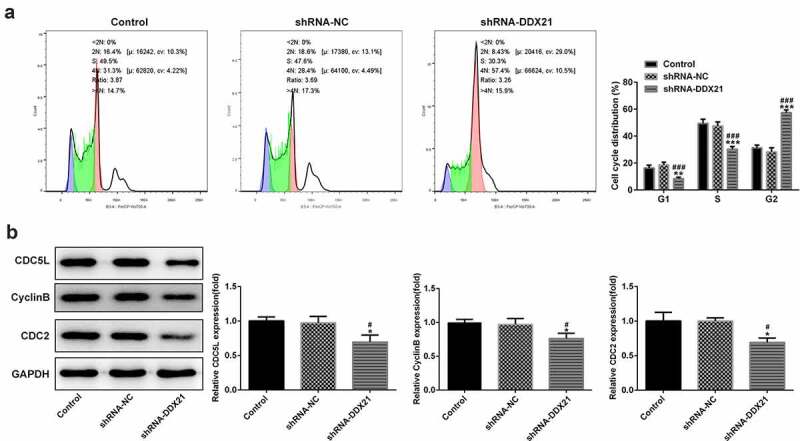
DDX21 knockdown inhibits G2/M transition. (a) Cell cycle was determined by flow cytometry (**p < 0.01, ***p < 0.001 vs. control; ^###^p < 0.001 vs. shRNA-NC). (b) The expression of CDC5L, CyclinB and CDC2 in different groups (*p < 0.05 vs. control; ^#^p < 0.05 vs. shRNA-NC)

Given the increasing levels of DDX21 in CRC cells, we sought to investigate whether DDX21 participates in CRC cell proliferation. DDX21 was knocked down in the SW480 cells and the transfection efficiency was evaluated by RT-qPCR (Figure 2a). The knockdown efficiency of shRNA-DDX21-1 was better thus shRNA-DDX21-1 was used for further experiments (Named as shRNA-DDX21). Cells proliferation was prominently decreased following DDX21 knockdown detected by CCK-8 and clone formation assay (Figure 2b,c). Moreover, immunofluorescence assay showed that the proliferating cell nuclear antigen ki-67, was expressed lower in DDX21 knockdown cells than in control or shRNA-NC group (Figure 2d). In cell cycle analysis, the cell number reduction in G1 and S phase was coincided with an increase of G2/M phase (Figure 3a). The G2/M phase marker cyclin B and CDC2 expressed less in DDX21 knockdown cells than in normal cells (Figure 3b). The levels of CDC5L, also known to regulate G2 phase transfer to M phase, was expressed lowly following DDX21 knockdown (Figure 3b). In short, the data exhibit that DDX21 is needed in tumor cell proliferation and cell cycle of CRC cells.

## DDX21 knockdown inhibit tumor growth for CRC in vivo

**Figure 4.**
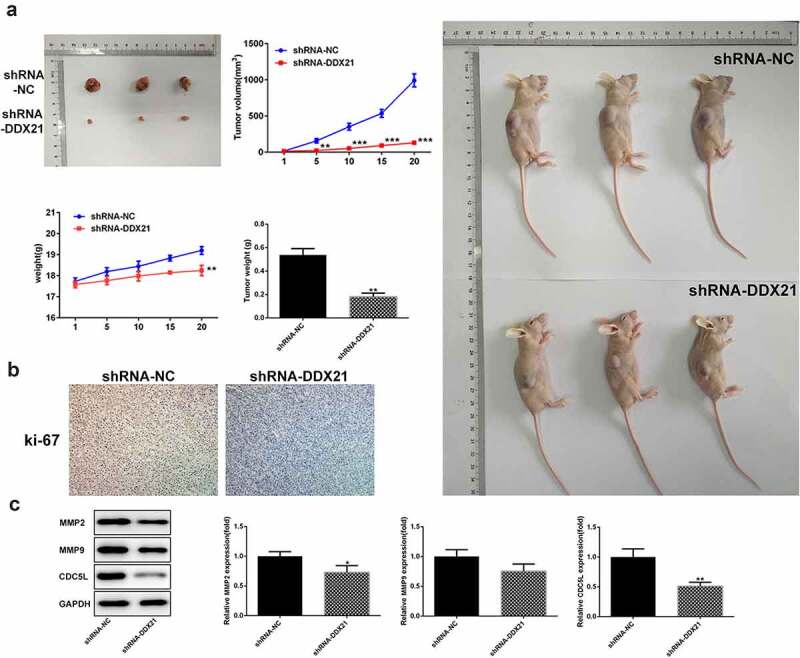
DDX21 knockdown suppressed tumor growth. (a) The role of DDX21 on tumor growth was assessed in tumor xenograft *in vivo* models (**p < 0.01 vs. shRNA-NC). (b) The expression of ki-67 in tumor tissues by immunohistochemical. (c) The expression of MMP2, MMP9 and CDC5L in tumor tissues (*p < 0.05, **p < 0.01 vs. shRNA-NC)

To estimate the long-term roles of DDX21 deficiency on tumor proliferation, we implanted the DDX21 knockdown cells in BALB/c nude mice. After 20 days, the three tumors in shRNA-NC mice developed steadily elevating that were higher than the three in shRNA-DDX21 mice (Figure 4a). Moreover, knock-down of DDX21 decreased the expression levels of a proliferative marker, ki-67 (Figure 4b). The results showed that DDX21 was crucial in CRC tumor growth *in vivo*. We also found that CDC5L was decreased following DDX21 knockdown (Figure 4c). In addition, MMP2 and MMP9 expression were decreased in cells with DDX21 knockdown revealing that DDX21 might regulate CRC migration (Figure 4c).

## DDX21 targets CDC5L directly

**Figure 5.**
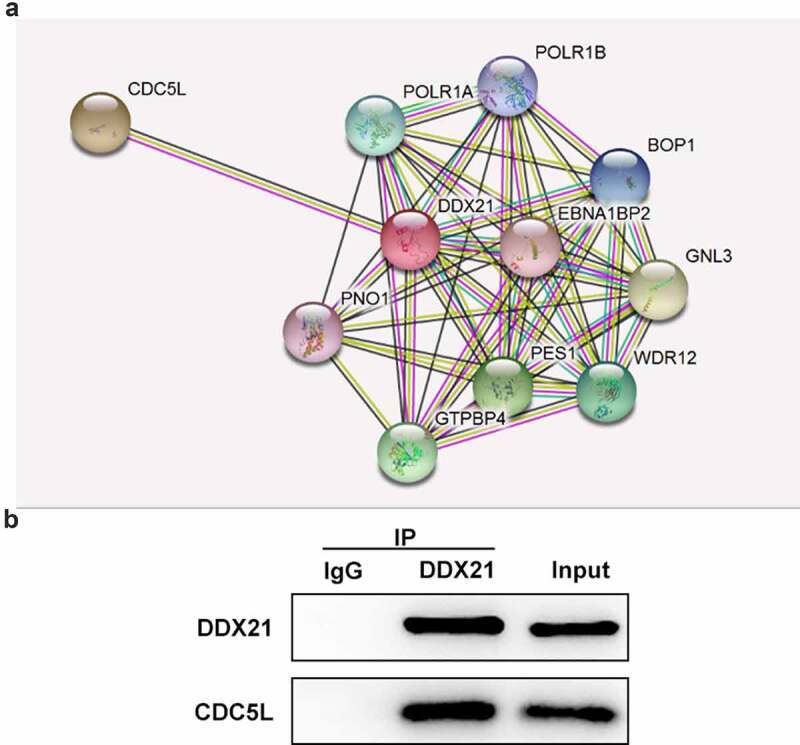
DDX21 interacted with CDC5L. (a) The prediction of interaction of DDX21 and CDC5L by online database String. (b) The interaction of DDX21 and CDC5L was assessed by Immunoprecipitation

To further investigate the potential mechanism underlying the regulation in DDX21 CRC, We used the STRING online database to predict the target genes of DDX21. CDC5L was found to be a potential target of DDX21 (Figure 5a). From data in Figure 1b and C, CDC5L was highly expressed in CRC cell lines. In addition, Co-IP assay indicated that DDX21 interacts with CDC5L (Figure 5b).

## DDX21 regulates the progression of CRC by interacting with CDC5L

**Figure 6.**
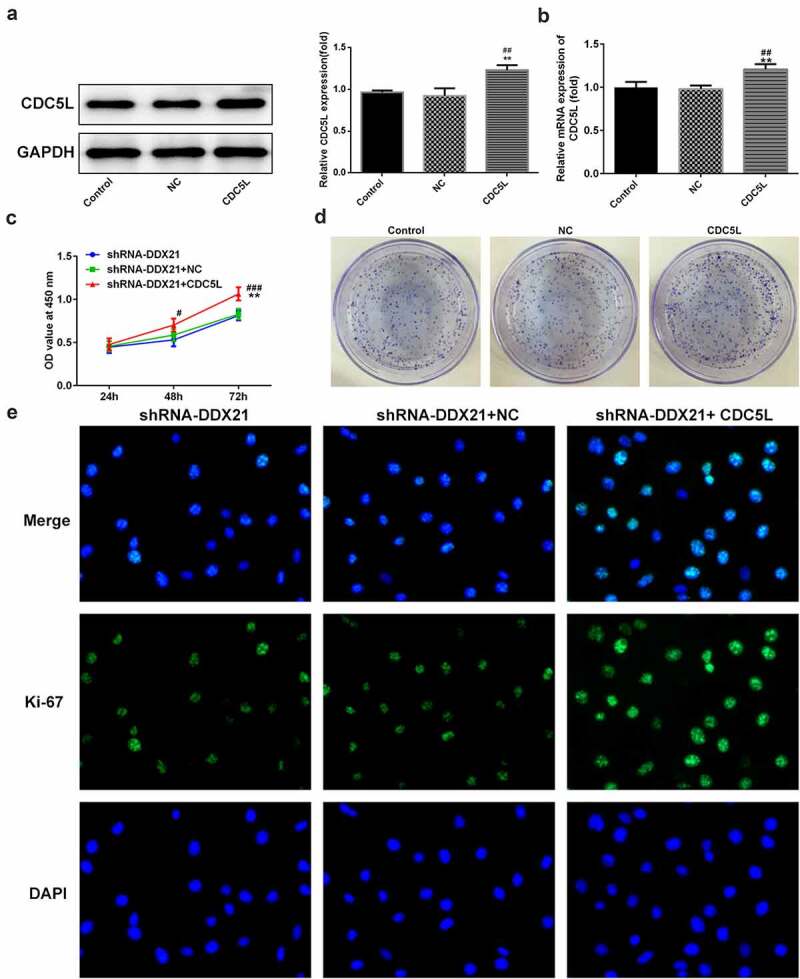
The cell proliferation was higher in SW480 (CDC5L-shRNA-DDX21) cells than in SW480 (shRNA-DDX21) cells. (a-b) The expression of CDC5L in SW480 (sh-RNA-DDX21) control group, and SW480 (shRNA-DDX21- pcDNA3.1 empty control) NC group, and SW480 (CDC5L-shRNA-DDX21) CDC5L group was determined by Western blots and RT-PCR, respectively (**p < 0.01 vs. control; ^##^P < 0.01 vs. NC). (c) Cell viability was detected by CCK-8 at 24, 48, 72 h (**p < 0.01 vs. shRNA-DDX21, ^#^P < 0.05, ^###^P < 0.001 vs. shRNA-DDX21+ NC). (d) The proliferation of SW480 cells in different groups was detected by clonal formation assay. (e) ki-67 expression and location in different groups was evaluated by immunofluorescence

**Figure 7.**
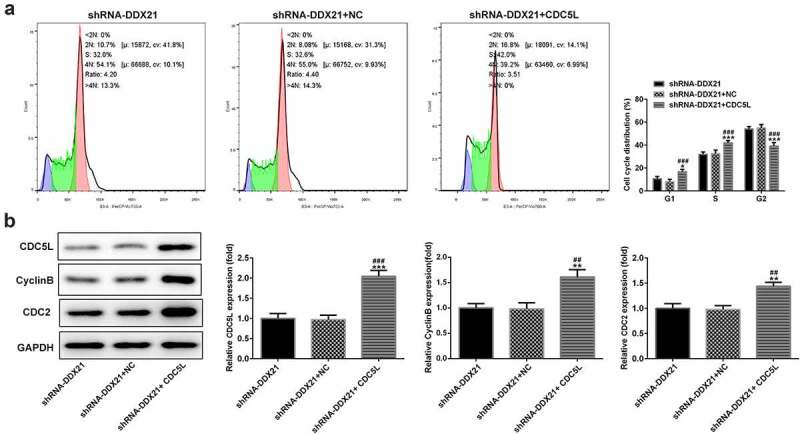
CDC5L promoted G2/M transition. (a) Cell cycle was determined by flow cytometry (*p < 0.05, ***p < 0.001 vs. shRNA-DDX21; ^###^p < 0.001 vs. shRNA-DDX21+ NC). (b) The expression of CDC5L, CyclinB and CDC2 in different groups (**p < 0.01, ***p < 0.001 vs. shRNA-DDX21; ^##^p < 0.01, ^###^p < 0.001 vs. shRNA-DDX21+ NC)

**Figure 8.**
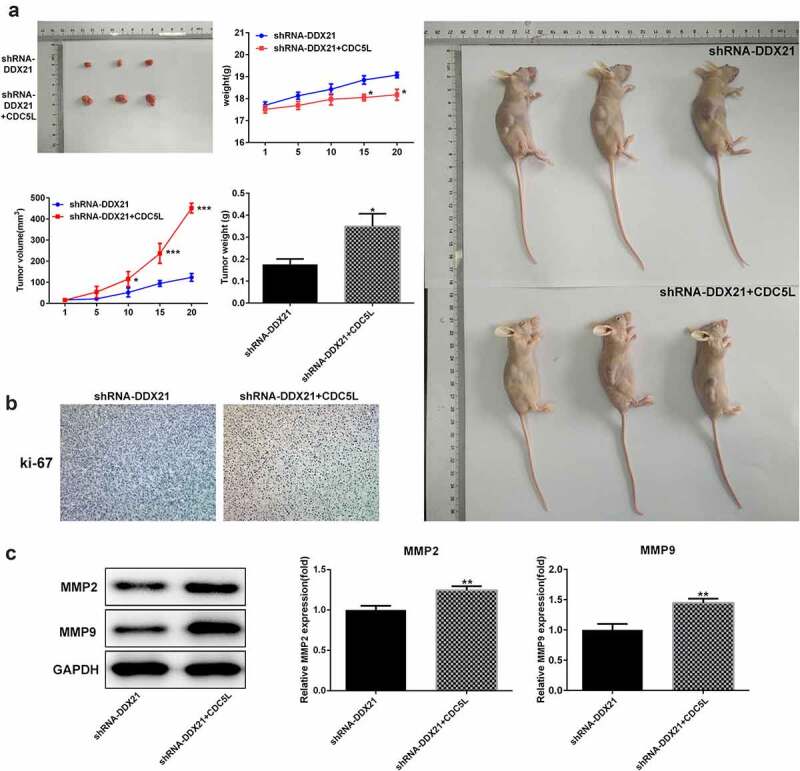
CDC5L enhanced tumor growth. (a) The role of DDX21 on tumor growth was assessed in tumor xenograft *in vivo* models (*p < 0.05 vs. shRNA-DDX21). (b) The expression of ki-67 in tumor tissues by immunohistochemical. (c) The expression of MMP2, MMP9 and CDC5L in tumor tissues (**p < 0.01 vs. shRNA-DDX21)

Then, we evaluated whether DDX21 regulates cell proliferation and cell cycle via CDC5L in SW480 cells. We overexpressed CDC5L in DDX21-silenced SW480 cells and detected by RT-qPC R and Western blotting assay (Figure 6a,6b). Data showed that DDX21 silencing-mediated decreased cell proliferation was increased after overexpressed CDC5L in cells transfected with shRNA-DDX21 (Figure 6c,6d). In addition, ki-67 was increased dramatically after CDC5L overexpression (Figure 6e). The results from flow cytometry indicated that the G2/M transition was promoted by overexpression of CDC5L (Figure 7a). In addition, Western blot assay also showed that the protein expression of CDC5L, CyclinB and CDC2 were enhanced by CDC5L overexpression. (Figure 7b). Then, we injected the SW480 cells with different treatments into the mice. Data showed that overexpression of CDC5L increased the tumor growth in DDX21 knockdown tumors (Figure 8a), and ki-67 expression was significantly increased in sh-DDX21-CDC5L tumors (Figure 8b) Besides, MMP2 and MMP9 expression was also increased by CDC5L (Figure 8c). These results suggest that DDX21 may play regulatory roles in the progression of CRC partly by interacting with CDC5L.

## Discussion

The survival rate of CRC in worldwide is still low [26]. Although the investigation for etiology and pathogenesis of CRC is processing, the molecular mechanisms are still rarely known. Chemotherapy is the most commonly used in CRC therapy, but the severely side effects of chemotherapy limit the use and drives us to develop new alternative targets [27].

The current investigation found that DDX21 was dramatically overexpressed in certain CRC cells, but the role and special mechanism of DDX21 in CRC are not reported yet [28]. Thus, we firstly assessed the role of DDX21 both *in vitro* and *in vivo*. In this study, downregulation of DDX21 promoted the cell cycle arrest and subsequently suppressed the cell proliferation. After xenograft with shRNA-DDX21 SW480 cells or wild-type SW480 cells, the tumor growth was detected. Also, the tumor growth was reduced after inhibition of DDX21 *in vivo*. Except for the growth of tumors, we detected the expression of matrix metalloproteinase (MMP) −2 and −9, and they were downregulated in DDX21 knockdown tumors compared with normal control indicating the potential role of DDX21 on tumor metastasis in CRC; however, data not shown the tumor metastasis in this experimental condition. DDX21 has been reported to negatively regulate Snail and subsequently epithelial–mesenchymal transition and metastasis in breast cancer [29]. It is a valid strategy to block the mitotic stage for cancer therapy. The alteration of MMP-2 and −9 reconstructs the tumor microenvironment and regulates the tumor metastasis [30]. A few investigations found that DDX21 enhances the expression of cyclinD1 and CDK2 and promotes cell growth in gastric cancer [5]. DDX21 has been reported as an RNA helicase to regulate cell growth [6]. Thus, DDX21 can be an effective therapeutic target for colorectal cancer.

In the study, CDC5L was highly expressed in CRC cells, which has been reported to promote G2/M transition in cell cycle [31]. DDX21 silencing reduced the expression levels of CDC5L both *in vitro* and *in vivo*. In vitro analysis also showed that DDX21 promoted G2/M phase arrest, as well as the downregulation of G2/M biomarker, cyclin B and CDC2. Based on the results, DDX21 may play roles on cell cycle mediated by CDC5L. To further confirm the hypothesis, we used an online STRING database and predicted that DDX21 interacts with CDC5L. We first proved that DDX21 did interact with the CDC5L detected by immunoprecipitation. Besides, we found that overexpression CDC5L in SW480 (shRNA-DDX21) cells reversed the inhibition of cell proliferation, cell cycle arrest or tumor growth, which indicate the involvement of CDC5L in regulatory effects of of DDX21 on CRC development. However, there are several limitations in our current study. Because we mainly focused on the target and mechanism by which DDX21 regulates CRC, we only used one CRC cell line to explore the role and mechanism of DDX21. We will verify our results in more CRC cell lines, explored wider biological effects of DDX21 in other CRC cell lines and investigating other potential mechanism in our next study. In addition, we found that DDX21 has the promising role in tumor metastasis in CRC; however, how DDX21 exert its role in tumor growth of mice is still need to be explored.

## Conclusions

Our data mainly showed that DDX21 is a tumor promoter in CRC. Downregulation the DDX21 effectively hindered cell proliferation and cell cycle, as well as the tumor growth. In addition, DDX21 may regulate CRC by interacting with CDC5L, which may provide a novel fundamental insight into pathogenesis of CRC and identify new therapeutic targets for CRC patients.

## Highlights

- DDX21 silencing inhibits cell proliferation and blocks cell cycle in CRC cells.
- DDX21 silencing represses tumor growth and metastasis in xenograft mouse model.
- DDX21 binds to CDC5L directly.
- DDX21 regulates CRC development by targeting CDC5L.
